# Towards an Automated Unsupervised Mobility Assessment for Older People Based on Inertial TUG Measurements

**DOI:** 10.3390/s18103310

**Published:** 2018-10-02

**Authors:** Sandra Hellmers, Babak Izadpanah, Lena Dasenbrock, Rebecca Diekmann, Jürgen M. Bauer, Andreas Hein, Sebastian Fudickar

**Affiliations:** 1 Carl von Ossietzky University Oldenburg, 26129 Oldenburg, Germany; babak_izadpanah83@yahoo.com (B.I.); rebecca.diekmann@uni-oldenburg.de (R.D.); andreas.hein@uni-oldenburg.de (A.H.); sebastian.fudickar@uni-oldenburg.de (S.F.); 2 Peter L. Reichertz Institute for Medical Informatics, 30625 Hannover, Germany; lena.dasenbrock@plri.de; 3 Center for Geriatric Medicine, University Heidelberg, 69117 Heidelberg, Germany; Juergen.Bauer@bethanien-heidelberg.de

**Keywords:** TUG, IMU, frailty, geriatric assessment, machine learning, wearable sensors, semi-unsupervised, self-assessment, domestic environment, functional decline

## Abstract

One of the most common assessments for the mobility of older people is the Timed Up and Go test (TUG). Due to its sensitivity regarding the indication of Parkinson’s disease (PD) or increased fall risk in elderly people, this assessment test becomes increasingly relevant, should be automated and should become applicable for unsupervised self-assessments to enable regular examinations of the functional status. With Inertial Measurement Units (IMU) being well suited for automated analyses, we evaluate an IMU-based analysis-system, which automatically detects the TUG execution via machine learning and calculates the test duration. as well as the duration of its single components. The complete TUG was classified with an accuracy of 96% via a rule-based model in a study with 157 participants aged over 70 years. A comparison between the TUG durations determined by IMU and criterion standard measurements (stopwatch and automated/ambient TUG (aTUG) system) showed significant correlations of 0.97 and 0.99, respectively. The classification of the instrumented TUG (iTUG)-components achieved accuracies over 96%, as well. Additionally, the system’s suitability for self-assessments was investigated within a semi-unsupervised situation where a similar movement sequence to the TUG was executed. This preliminary analysis confirmed that the self-selected speed correlates moderately with the speed in the test situation, but differed significantly from each other.

## 1. Introduction

**Figure 1:**
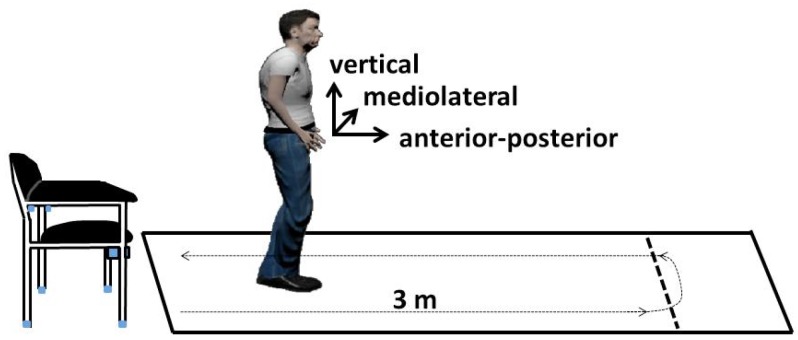
Setting of the Timed up and Go (TUG) test in our study. The test is measured by an IMU integrated into a belt. Additionally, a stopwatch and the automated/ambient TUG (aTUG) system are reference measures. The coordinate orientation of the Inertial Measurement Unit (IMU) is illustrated in the figure.

The early detection of functional decline, which occurs with age and results, among others, in an increased fall risk, is important to initiate timely preventive measures, slow down the progress of decline and maintain older peoples’ independent living. The Timed Up and Go test (TUG), developed by Podsiadlo and Richardson [1], is a well-established assessment and one of the most frequently-used tests for mobility. This test assesses the basic mobility skills, which make up important abilities for independent living. Besides balance and strength, mobility is one of the essential components of physical function [2]. The TUG consists of several components of everyday movements (see Figure 1). At the beginning of the test, the patient is sitting on a chair leaning against the backrest. Then, she or he gets up from the chair, walks 3 m, turns around, walks back and sits down again. The test starts with the request and stops when the participant is seated again correctly in the chair with his/her back resting at the back of the chair. According to the necessary duration, the patient’s mobility is categorized into four groups: <10 s freely mobile, 11–19 s mostly independent, 20–29 s variable mobility, >30 s impaired mobility. Therefore, a faster time indicates a better functional performance. The TUG test is an established and widely-used test as part of geriatric assessments, and the intra-rater, inter-rater and test-retest reliability of the individual components of the instrumented TUG (iTUG) have been shown to be excellent to good for total duration in patients with Parkinson’s disease [3]. Therefore, the TUG test is a suitable test to investigate temporal progressions. For example, van Iersel et al. [4] pointed out that the TUG test is sensitive to clinically-relevant changes in functional mobility in frail elderly patients within a period of two weeks. Over time, the TUG test was enhanced several times: Wall et al. [5] introduced the Expanded Timed Get-up-and-Go (ETGUG) test, in which the length was increased to 10 m, and times for each of the components tasks were measured separately using a multi-memory stopwatch.

The advantage of the TUG test is its simplicity: the short test duration, the low required equipment and the possibility to perform the test even for patients with functional impairments. However, because of the importance of detecting early changes in functional decline, these assessment should be performed regularly and in a high frequency to initiate early interventions and slow down the functional decline. To relieve clinicians and reduce the stress for the patients, these assessments should take place in the daily life of the patients in the form of unsupervised self-assessments. Inertial Measurement Units (IMU) are well suited for automated analyses and can be easy to use, flexible and inexpensive, thus suitable for self-guided assessments.

Sprint et al. [6] provided in their survey an overview of the TUG test and technologies utilized for TUG instrumentation. Besides video-based measurements, ambient technologies and wearable or smartphone-based technologies have been already used for studies. The automated/ambient TUG (aTUG) [7] uses ambient sensors attached to a chair and provides a fully-automated TUG test execution. The aTUG consists of a laser range scanner for gait analysis, force sensors in the chair legs to analyze the components of rising from the chair or sitting down and a laser barrier to measure the test duration, which shows a high correlation with stop-watch measurements [8].

The suitability of the aTUG system in diverse health care environments was already shown in [9].

Wearable sensors have become quite popular, and therefore, IMU were used in several studies. Table 1 shows a selection of studies, which used inertial measurement units or smartphones for TUG analyses.

Already in 2008, Higashi et al. [10] developed the TUG-T and measured the duration of the six single components of the test (standing up, walking forward, turn one, walking back, turn two and sitting down) with two inertial sensors. An instrumented version of the TUG (iTUG) was proposed to measure the duration of four components (sit-to-stand, steady-state gait, turning, turn-to-sit) and a set of balance and gait parameters [11], but required seven inertial sensors. Reinfelder et al. [16] developed a TUG phase segmentation system with two IMU and reached a mean sensitivity of 81.80% over all phases by using a support vector machine. A smartphone application called sTUG was developed by Milosevic et al. [15], which completely automates the iTUG test, determines the beginning and the end of the test and quantifies its individual phases. With a system of 17 IMU and a rule-based algorithm, accuracies of 100% could be achieved for TUG phases’ recognition [17].

Many studies in the literature focus on the discrimination between different diseases or phenotypes such as Parkinson’s disease or fallers like for example [12,18]. Although there are good results for combined use of multiple IMU, the recognition accuracy of the TUG phases with a single IMU can be improved and should be confirmed for larger study populations. Many studies used rule-based algorithms, while some others used logistic regression models for fall prediction [19], dynamic time warping [14] or for example feature selection [12]. Until now, only a rather small minority use machine learning for the TUG classification like [16]. However, in order to support a higher motion variety and motion anomalies, machine learning might be more suitable regarding different study populations, which differ for example in age, functional status or diseases, which affect the locomotor system. Additionally, these studies do not focus on settings beyond the laboratory, as well as automated self-assessments, except Milosevic et al. [15]. However, Milosevic’s system needs the interaction of the user (start and stop record) since it cannot identify phases of the TUG execution automatically in a longer sequence.

Thus, we develop an activity recognition via machine learning and a rule-based algorithm, which detects both standardized TUG-sequences and similar sequences, which may occur in daily life and is more naturalistic than the standard TUG setup. We used one single IMU integrated into a belt to enable an easy use and an unobtrusive sensor placement, especially regarding future self-assessments at home. After the validation of our system under standardized conditions in a laboratory environment, we evaluate the suitability for future self-assessments by a non-standardized situation with similar movement patterns to the TUG test.

In contrast to the previous studies, we conducted a study with a larger sample size to cover common age-related varieties of motion patterns and corresponding anomalies. As the ground truth, we applied both clinical caregivers (stopwatch measurements) and the aTUG system. In comparison to Milosevic’s system, we chose a more unobtrusive and easier sensor placement than the chest. Our system can be used without assistance and interactions. Another major difference lies in the application of a machine learning algorithm instead of a rule-based algorithm. Since a high variance in movement patterns or strategies can occur, especially for transitions, in older adults, machine learning algorithms might be more robust than rule-based algorithms. If large datasets are available, which cover a majority of these variances, it is preferable to use machine learning. Rule-based algorithms need to be very complex to take different study populations into account, which differing in functional status or diseases. With increasing complexity, reading and adjusting a rule-based system becomes often cumbersome.

In summary, we want to develop an accurate system for automated mobility assessments for older people based on inertial TUG measurements, which is able to be used in clinical environments, as well as in future unsupervised self-assessments at home for a high variety of patients.

## 2. Materials and Methods

**Figure 2:**
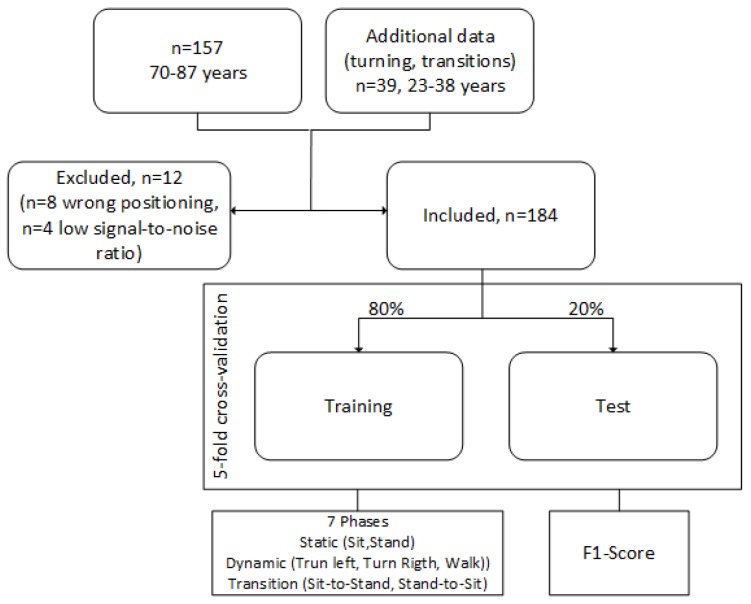
Used data for the machine learning model. Additional data of a younger study population (*n* = 20, aged 23–37 years) was included for optimization of the recognition of turnings and transitions.

In order to evaluate an approach for automated measurement and analysis of the TUG test based on 3D accelerometer and 3D gyroscope data via machine learning, we included 157 participants in our study (87 female (59%), 60 male (41%)), aged 70 years and above. Table 2 lists the characteristics of our study population. The data of 10 participants were excluded in this analysis, due to a low signal-to-noise ratio or a wrong positioning of the sensor (cf. also Figure 2).

Besides the TUG test, the screening study consists of other different geriatric tests such as the Short Physical Performance Battery (SPPB), the Stair Climb Power Test (SCPT), the 6-min walk test, frailty criteria and counter movement lump. These tests were measured in a conventional way by medical professionals and additionally with ambient and wearable technology. More details are described in [20]. The study has been approved by the appropriate ethics committees (ethical vote: Hannover Medical School, No. 6948; ethical vote: Carl von Ossietzky University, Drs.33/2016) and conducted in accordance with the Declaration of Helsinki.

We used a five-fold cross-validation. Therefore, our dataset was randomly divided into 5 equally-sized subsamples. One subsample (20% of the data) was retained as the validation data for testing the model, and the remaining 4 subsamples (80%) were used as training data. The cross-validation process was then repeated 5 times, with each of the 5 subsamples used once as the validation data. Figure 2 illustrates the data used for our machine learning model. Due to optimization steps and to enable an increased sensitivity, we added additional data of a younger study population (*n* = 39, 23–38 years) for left and right turnings, as well as sit-to-stand and stand-to-sit transitions, which were underrepresented in our dataset. Imbalanced data refer often to classification problems because standard classifiers tend to be overwhelmed by the large classes and ignore the small ones [21]. Before this optimization step, data of these activities were underrepresented in comparison to walking and sitting.

The geriatric tests in our study were supported by technology. Thereby, the TUG was technically measured via the aTUG system and the sensor belt, which are described in the following.

### 2.1. aTUG System

**Figure 3:**
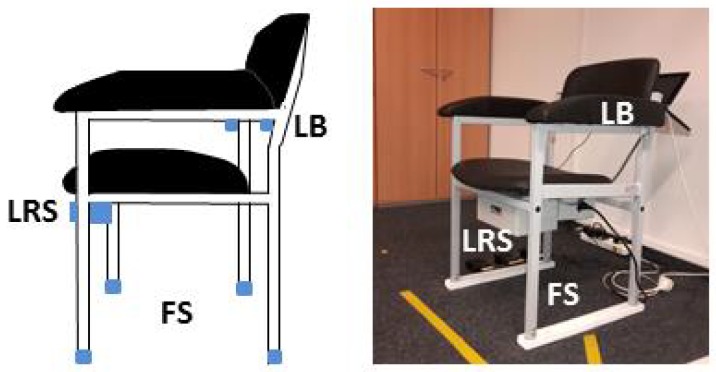
The aTUG system is used for automated TUG tests and includes force sensors (FS) in each chair leg, a laser range scanner (LRS) and a light barrier (LB).

The aTUG system is illustrated in Figure 3. It includes four force sensors (FS) in each chair leg, a laser range scanner (LRS) and a light barrier (LB). The force sensors (rated force: 1 kN, accuracy class: 1%) measure the force distribution on the chair. Especially the transitions (sit-to-stand, stand-to-sit) can be analyzed by these sensors. The TUG duration analysis by force sensors achieved a root mean square error (RMSE) of 0.90 s after calibration [8]. The laser range scanner (Hokuyo UTM-30LX, Hokuyo Automatic Co., Ltd., Osaka, Japan) was used for gait analyses during the walking phases and the turning. Additionally, a light barrier (OSRAM LD271, OSRAM Opto Semiconductors GmbH, Regensburg, Germany) was mounted at the backrest of the chair to detect the beginning and end of the TUG test. All sensors are commercially available. The aTUG system is able to detect the duration of the TUG test with a mean error of 0.05 s and a standard deviation of 0.59 s [22].

Due to its valid and precise measurements, the aTUG system is used as the reference system.

### 2.2. Sensor-Belt

**Figure 4:**
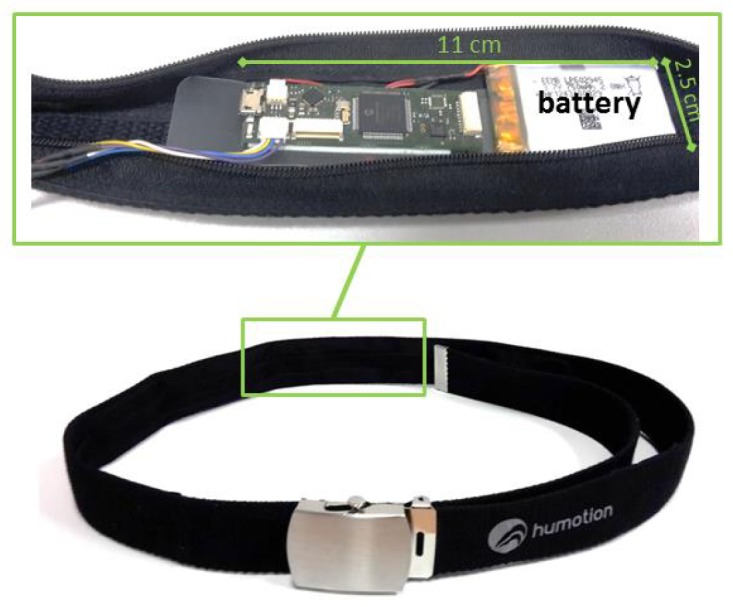
The sensor belt includes a 3D accelerometer, gyroscope and magnetometer, as well as a barometer.

Besides the aTUG system, a wearable system was utilized, which is also commercially available. Figure 4 shows the sensor system, which is integrated into a belt and worn at the hip. The dimension of the sensor unit is about 11 cm × 2.5 cm (battery included), and the overall weight of the belt is 140 g. This compact and light system enables an easy, unobtrusive and comfortable measurement. The sensor unit consists of a triaxial accelerometer (Bosch BMA180, Bosch Sensortec GmbH, Reutlingen, Germany), gyroscope (STMicroelectronics L3GD20H, STMicroelectronics, Geneva, Switzerland) and magnetometer, as well as a barometer. The sensitivity of the accelerometer is ±16G and the resolution 12 bit, while the sensitivity of the gyroscope lies at ±2000 deg · $s^{-1}$. We used a general sampling rate of 100 Hz in our study. The orientation of the sensors is illustrated in Figure 1. The correct placement of the sensor belt, as well as the position of the sensor unit inside the belt were checked for each participant of our study by our physical therapists or study nurses and was adapted individually to ensure a correct alignment between the L3 and L5 lumbar vertebral body. Especially regarding our machine learning approach, a correct alignment is important for a good classification performance.

In our study, we used only the inertial sensors (accelerometer and gyroscope) to avoid over-fitting, because the magnetometer is highly influenced by environmental noise (metal chair). The barometer can be used to detect changes in height. However, since the accuracy lies about ±10 cm, the air pressure data were excluded due to their low additional information content.

### 2.3. Machine Learning and Algorithm

#### 2.3.1. Pattern of TUG Test and Labeling

**Figure 5:**
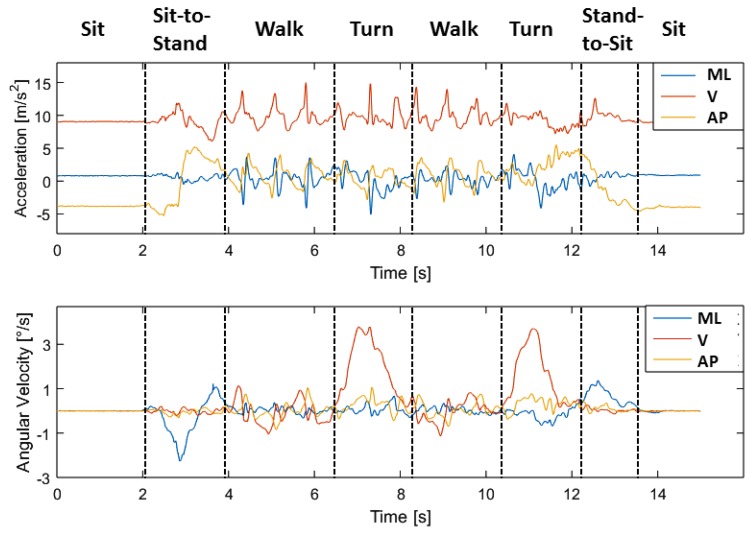
Example of the acceleration and gyroscope data during a TUG test. The TUG test consists of several components of everyday movements, which are marked in the graph. Each component is characterized by specific features, which are derived for machine learning classification. Medio-Lateral (ML); Vertical (V); Anterior-Posterior (AP).

Figure 5 shows exemplarily the acceleration and gyroscope data of one person in three axes (vertical, mediolateral and anterior-posterior) during the TUG test. The coordinate orientation of the sensors is illustrated in Figure 1. The different phases of the TUG are marked in Figure 5. The static activity of sitting at the beginning and the end of the TUG can be easily recognized based on its nearly constant values of acceleration and angular velocity. Especially for the phases of turning, the gyroscope data in y-direction show significant peaks. The walking phases are characterized by peaks, which can be used for step detection. In the shown example are three steps before the participants starts the turning. The overall duration of this tests is about 12 s (14−2 s). The decision between turning and stand-to-sit at the end of the TUG test can be difficult, because of a possible overlay of these movements.

Supervised learning is a type of machine learning algorithm that uses a known dataset to make predictions for a new dataset. To create this training set, the TUG-phases are manually labeled by experts regarding their acceleration and gyroscope signals via a rule-based method. After labeling, features are derived for classification for each movement. In order to describe our algorithm, we will focus on the derived features, the sliding window and the classifier in the next subsection.

#### 2.3.2. Hierarchical Classification Model

**Figure 6:**
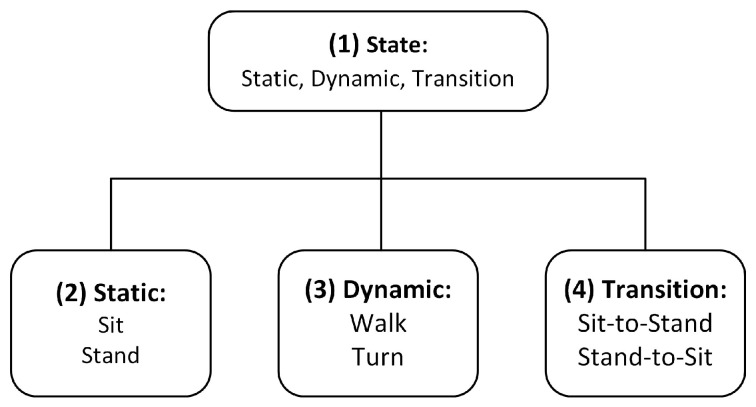
Hierarchical classification model. The first classifier distinguished between the state, and the others classify the possible activities of each state.

We developed a hierarchical model for classification (see Figure 6). Therefore, four classifiers were trained. After a low pass filtering of the raw data, the first classifier (1) distinguished between static and dynamic activities, as well as transitions. If the state was classified, the other classifiers (2)–(4) characterized the activities in detail after filtering the raw data with the mentioned filters in Table 3.

The features of the specific phases are extracted to characterize the movements. The used features are:
• Auto Correlation (AC)• Mean (M)• Pitch (P)• Standard Deviation (SD)• Root Mean Square (RMS)• Signal Energy (SE)• Signal Magnitude Area (SMA)• Signal Vector Magnitude (SVM)• Spectral Entropy (SE)• Correlation (C)

A detailed description of each feature and its calculation can be found in [23].

Since activity recognition requires a careful selection of feature combinations for classification, the number of features used is limited, so that only these are used, which significantly improves the classification model. This is done because, on the one hand, not every feature combination is suitable for each classification and, on the other hand, the computation time must remain efficient.

We used four different classification models to optimize our activity recognition: Boosted Decision Trees (BDT) [24,25], Boosted Decision Stump (BDS) [26], Multilayer Perceptrons (MLP) [27] and Adaptive Multi-Hyperplane Machine (AMM) [28]. The *F*1-score, recall, precision and accuracy are used for validations: the *F*1-score is the harmonic mean of precision, and recall and is defined by:

(1) $$F1=2\cdot \frac{precision\cdot recall}{precision+recall},$$

where precision and recall are defined by:

(2) $$precision=\frac{tp}{tp+fp}$$

and:

(3) $$recall=\frac{tp}{tp+fn},$$

with *tp* as true positive, *fp* as false positive and *fn* as false negative. The accuracy is defined by:

(4) $$accuracy=\frac{tp+tn}{tp+tn+fp+fn},$$

with *tn* as true negative.

Table 3 sums up the parameters for our classifiers, which were determined after optimization analyses.

The methods, as well as the sliding window parameters and the feature sets are different for each classifier. The combinations with the best results are presented here (cf. Table 4). Thereby, the features were calculated in each case for all components of Acceleration (Acc), Gyroscope (Gyro) or for both (Acc + Gyro).

### 2.4. TUG Analyses Algorithm

The raw data are classified via our hierarchical classification model as described in Section 2.3.2. Since we want to detect complete TUG sequences, we used a rule-based model to identify the TUG test via its specific phases and their order. For this, the order of the automatically generated labels of the classified sub-activities will be checked. A valid TUG sequence consists of the following phases (see Figure 1 and Figure 5):

Sit-to-stand → walk → turn → walk → turn → stand-to-sit.

Since the sub-activities can be expected to include some classification errors, due to the false-positive rates of the trained classifiers (see Table 4), and to take the variations of performing the TUG test into account, we included the following approach to increase the robustness of the algorithm. Nine models were specified as valid TUG sequences. Especially, doubled activities such as turn-turn were accepted as valid and were combined as one turn. Another valid model consisted of the combination sit-to-stand → stand-to-sit → walk, which is implausible and indicates a misclassification. Therefore, our model allows minor illogical classification errors. For example:

- Sit-to-stand → stand-to-sit → walk → turn → turn → walk → turn → stand-to-sit
- Sit-to-stand → walk → turn → turn → walk → turn → stand-to-sit
- …

Especially regarding the aim to analyze further unsupervised-assessments in non-standardized settings, this more robust approach might be more applicable. A list of all models and their accuracies is presented in the Results Section 3.3. The duration of each phase can be determined by these motion labels, as well as the overall duration of the TUG test. In the evaluationsection, we compare our results with stopwatch measurements by medical experts and the aTUG system.

## 3. Results

After the description of the study design, sensors used and the algorithms, we want to focus on our results in the following.

### 3.1. Results of the Hierarchical Classification Model

Table 4 shows the results of the *F*1-scores for the best combination of each method. While boosted decision trees achieved the highest *F*1-score for the state-classification, multilayer perceptrons were most suitable for the classification of static and dynamic activities, as well as transitions.

### 3.2. Results of TUG-Phases’ Classification

The results for recall, precision, accuracy and *F*1-score for our classification of each TUG component are listed in Table 5. We achieved *F*1-scores >0.94 for the static activities standing and sitting, as well as for the dynamic activity walking. However, especially for the short movements such as the transitions and turnings, we only achieved *F*1-scores between 0.70 and 0.81. For further optimization, more data of these activities are needed, because these movements are still underrepresented, even though we used additional data. However, accuracies above 0.96 could be achieved for all TUG components.

### 3.3. Results of TUG Classification

The TUG classification followed a rule-based model. The detected activities had to be in a specific order to be recognized as a valid TUG test. Since one of the valid models could be found in the data, the duration of the whole activity was calculated based on the durations of the single phases. The resulting duration had to be below 30 s to be accepted as TUG-test since durations over 30 s were not realistic because they failed our inclusion criteria.

As already mentioned in Section 2.4, nine models were accepted as valid TUG tests, to increase the robustness of our algorithm. The accuracy was estimated in accordance with Equation (4). The cumulative accuracy $A_{i}$ was calculated by the sum of the accuracies of the nine valid models $A_{j}$:

(5) $$A_{i}=\sum_{i=1}^{j}A_{j}$$

Table 6 lists the included models and the accuracies of TUG test detection.

The model in the first line corresponds to the standard sequence of TUG phases. With these nine considered models, we reach a recognition accuracy of 96.55%, which corresponds to the cumulative accuracy. Of course, more models can be created to achieve higher accuracies, but this would also lead to a higher false-positive rate.

### 3.4. Comparison with Stopwatch Measurement

**Figure 7:**
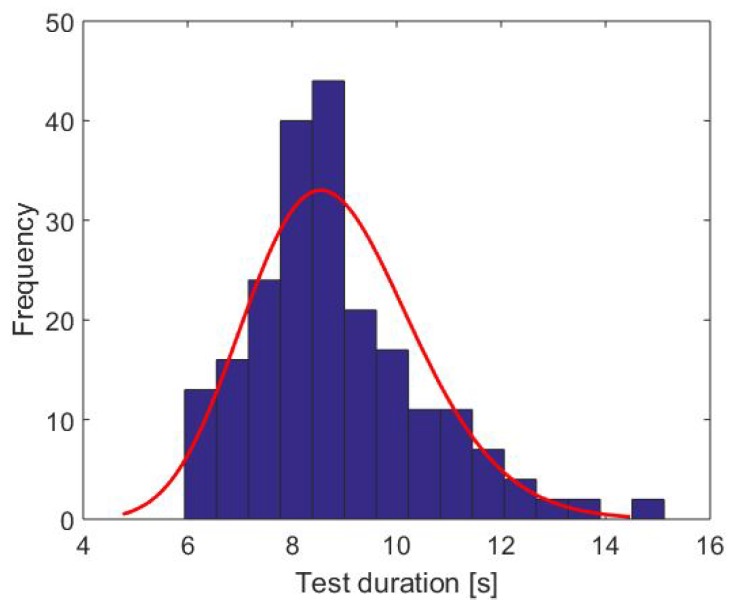
Distribution of the TUG test duration (stopwatch measurements) and the estimated gamma distribution (red line).

In order to validate our system regarding the measurement of the total time, we compared our results with two reference measurements, assumed as the gold standard. Besides the automated, technical measures, medical professionals measured the test duration by stopwatch. The histogram in Figure 7 shows the needed test duration for our study population (stopwatch measurements). Due to the left-skewed distribution, we estimated a gamma distribution (red line).

**Figure 8:**
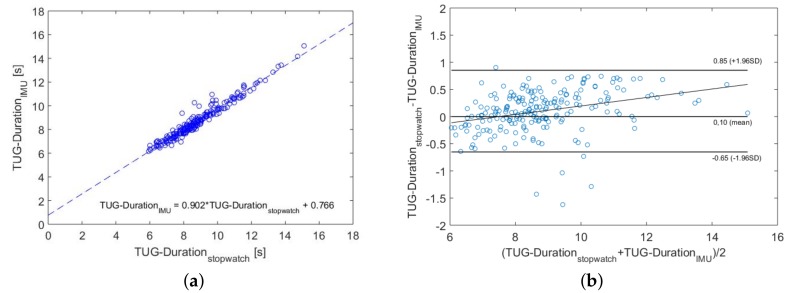
Comparison between stopwatch and IMU measurements. The dashed line represents the linear regression line and corresponds to the stated equation in (**a**). The Bland–Altman plot and its characteristic values are shown in (**b**). (**a**) Correlation analysis; (**b**) Bland–Altman plot.

A correlation analysis between the stopwatch and automated measurements showed a significant correlation with an excellent correlation coefficient of r=0.97 and a *p*-value of <0.001. Figure 8 refers to the comparison between the measured test duration by stopwatch and our IMU system. As expected, there was a linear relationship. A regression analysis results in the following relation:

(6) $$TUG\_Duration_{IMU}=0.90\cdot TUG\_Duration_{stopwatch}+0.766$$

Additionally, a Bland–Altman plot was used to analyze the agreement between both systems (see Figure 8b). Since the mean value was near zero, our IMU-based system had no fixed bias. The minimal detectable change differed in the literature between 1.14 s [29] and 3.4 s [30]. Therefore, differences within the mean ±1.96 SD were not clinically important, and the two methods may be used interchangeably.

### 3.5. Comparison with the aTUG System

**Figure 9:**
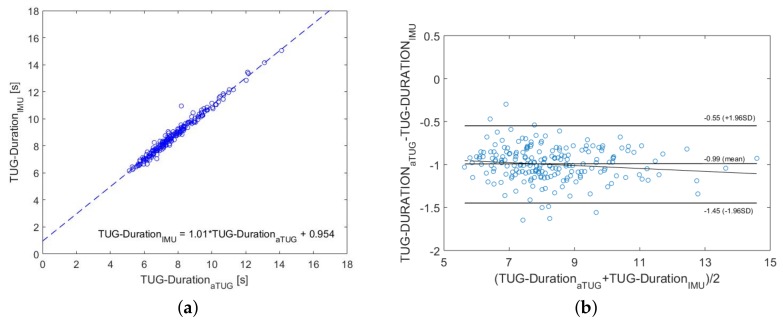
Comparison between aTUG and IMU measurements. The dashed line represents the linear regression line and corresponds to the stated equation in (**a**). The Bland–Altman plot and its characteristic values are shown in (**b**). (**a**) Correlation analysis; (**b**) Bland–Altman plot.

As already mentioned, we used the aTUG as an additional reference system. The correlation analysis has shown an excellent correlation coefficient of r=0.99 and a *p*-value of <0.001. Figure 9 shows the comparison between the aTUG and the IMU results. Again, there is a linear relationship:

(7) $$TUG\_Duration_{IMU}=1.01\cdot TUG\_Duration_{aTUG}+0.954$$

This was in good agreement with our previous findings. The marginally better correlation between aTUG and IMU than the stopwatch and IMU might be due to the inter-tester reliability, which influences the stopwatch measurements. Even though, these influences have been shown to be minimal [20].

The Bland–Altman plot indicated a consistent bias, which can be adjusted by subtracting the mean difference from the biased method. The differences within the mean 1.96 SD were not clinically important either. Therefore, these two methods can be also used interchangeably.

### 3.6. Suitability for Self-Assessments

**Figure 10:**
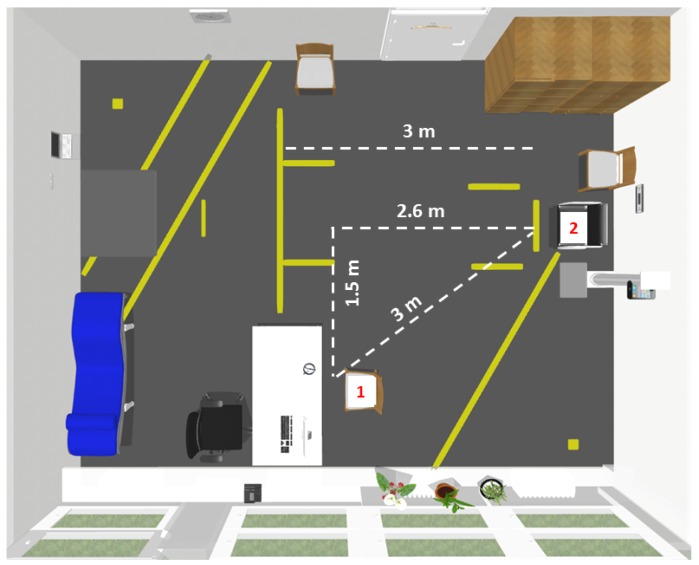
Sketch of our laboratory. Within a semi-unsupervised situation, the participants change from Chair 1 to Chair 2.

In order to evaluate the suitability of the sensor-belt for self-assessments, we asked the participants to wear the belt after the assessment for one week during the day. Since the battery runtime was about two days, the participants were instructed to load the battery every night, which worked well for most participants. Due to the individual positioning of the sensor-unit in the belt between the L3 and L5 lumbar vertebral body by the medical experts in the assessments, the sensor could also be positioned with a sufficient precision by the participants themselves. To confirm the suitability of our TUG classification for self-assessments, we classified the complete recordings of the test battery within the assessment, which consisted of several tests such as for example the short physical performance battery, the frailty criteria or the stair climb power test. Figure 10 shows our assessment room. To make an intermediate step to future home-assessments, we analyzed a semi-unsupervised situation. Within this test battery, the participants were asked to change seats (from Chair 1 to the aTUG-system (Chair 2)) as a preparation for the TUG-test (see Figure 10).

In this semi-unsupervised situation, the participants stood up from Chair 1 and walked with one turning to Chair 2 and sat down. This sequence was similar to a TUG sequence. The walking distances (white lines in Figure 10) varied between 3 m and about 4.1 m. Therefore, the mean distance was assumed to be 3.55 m.

**Figure 11:**
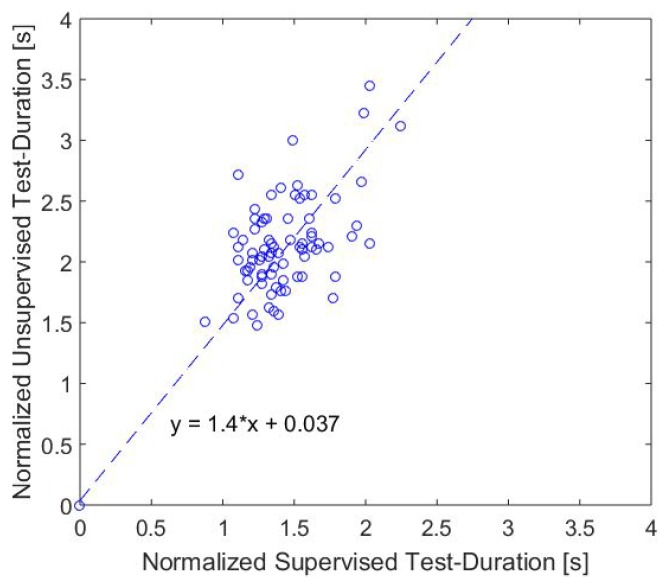
Comparison of the normalized durations of the TUG-test and the semi-unsupervised test situation.

Due to our set of valid TUG models, our system had the ability to recognize also these modified TUG sequences. Besides the already analyzed supervised TUG-tests, we identified 190 additional sequences in our data, which had a similar series of movements to the TUG test. In order to analyze this semi-unsupervised situation, we compared these results of 78 participants with the TUG-test duration. For better comparability, we normalized the data to the assumed walking distance (TUG-test: 6 m, change seats: 3.55 m). This approach had the limitation that the duration of the transfer movements (sit-to-stand, stand-to-sit) were supposed to be approximately the same, as well as the participants chose a mean distance of 3.55 m for changing seats. However, this first approximation showed a moderate correlation (r=0.51, p<0.01) between these parameters. Figure 11 shows the normalized test durations.

The results show that the self-selected speed was significantly slower than the speed in the test situation regarding the Wilcoxon test (p<0.01), which was applied due to the not normally distributed TUG test duration (see Figure 7). By assuming that the transfer movements and turnings were approximately the same, the differences in speed lied in the self-selected gait speed.

In order to investigate the correlation of the TUG test with the other battery of assessments, Pearson’s linear correlation coefficient was computed between the Standard TUG duration (STUG) and Unsupervised TUG duration (UTUG) with different geriatric tests. The results are listed in Table 7. The standardized TUG test had a higher correlation coefficient in all test than the semi-unsupervised TUG test.

## 4. Discussion

The main purpose of the present study was to develop an automated mobility assessments for older people based on inertial TUG measurements, which is able to be used in clinical environments, as well as in future unsupervised self-assessments at home. Therefore, we conducted a prospective study with 157 participants aged 70 and above and used the data to identify a suitable machine learning classifier for TUG-phases’ recognition. We achieved accuracies over 96% in the classification of the specific TUG components and for the complete TUG sequence recognition via a rule-based approach. In comparison to other studies, these results are satisfactory, especially regarding our minimalistic sensor system of one IMU, which was positioned in a belt at the hip. To validate our results, we compared the IMU-based data with two reference systems as the criterion standard, which showed significant correlations of 0.97 (stopwatch) and 0.99 (aTUG), respectively. This underlines the suitability of our system for clinical investigations. Thus, the system is a powerful, low-cost and accurate tool for automated TUG test analyses. Further analyses of the accuracy of our system to determine each TUG phase in terms of time, related to the reference system, should be done. This might be a valuable addition, especially for clinical issues, which addresses diseases with the influences of only specific phases of the TUG test.

Since we want to detect both standardized TUG-sequences and similar sequences, which may occur in daily life and are more naturalistic than the standard TUG setup, we focused as an intermediate step to unsupervised home-assessments on a semi-unsupervised situation with similar sequences to the TUG test within the assessment. The most important limitation and simultaneously a great opportunity lie in our set of valid TUG models. On the one hand, this could lead to misclassifications in laboratory settings, but on the other hand, it allows detecting movement sequences that are similar to the TUG test. The comparison of the durations confirmed the expected result that the self-selected speed is lower in non-test situations, but there is a moderate correlation between these tests. Maybe the self-selected speed can be more sensitive to functional changes than assessments in a test situation, when a participant tries to perform particularly well. However, in our first analyses, we could not confirm this hypothesis. Correlation analyses between different geriatric tests and the TUG variations show a stronger correlation between the standard TUG test and the other tests than for the semi-unsupervised TUG. This had been expected, since a stronger correlation was expected between the test situations.

The attraction and the novelty of our approach lies in the combination of a high accuracy in TUG classification with only one single inertial measurement unit, the large study population, the automated analyses, the simplicity of our the system (no interaction required, easy to use) and the suitability of the investigation of unsupervised or rather unstandardized assessments.

The general suitability of our sensor system was investigated during a home-assessment following the clinical assessments, in which the participants wore the sensor-belt during the day for one week and wrote an activity diary. Most participants were able to load the battery and wear the belt in the correct position by themselves.

Further analyses of the home-assessment data are planed to analyze the significance regarding the detection of functional decline. Especially, the investigation of the single TUG phases in unsupervised situations might be worthwhile. In summary, our system is applicable for flexible measurement of the Timed Up and Go test performance in clinical settings, as well as in semi-unsupervised situations. The determined IMU-based test durations are in good agreement with the stopwatch measurements by medical experts and the aTUG-system. Assessments of non-standardized variations of TUG sequences might be a worthwhile enhancement for the identification of changes in the functional status, but need further investigations.

## Figures and Tables

**Table 1:** Selection of studies that used inertial sensors for TUG analyses. The study population, as well as the placement of the IMU and the analyses method are listed in detail. Parkinson’s disease is abbreviated as PD; Accelerometer (Acc); Gyroscope (Gyro); Magnetometer (Magn); instrumented TUG (iTUG).

Article	Year	Population *n* (Years) (Average Age ± SD)	Technology, Placement	Methods, Analyses
Higashi et al. [10]	2008	10 healthy (21.0 ± 2), 20 hemiplegic (68.3 ± 11)	2 IMU (3D-Acc/Gyro), waist, upper thigh, video camera	duration of TUG phases, rule-based
Salarin et al. [11]	2010	12 PD (60.4 ± 8.5), 10 control (60.2 ± 8.2)	7 IMU forearms, shanks, thighs, sternum	automatic detection of TUG components, rule-based
Chiari [12]	2011	20 early-mild PD, 20 healthy	1 Acc lumbar segment L5	Feature selection, discrimination of PD and accuracy of 92.5%
Jallon et al. [13]	2011	19 subjects	1 IMU (3D-Acc), 3D-Magn, chest	Bayesian classifier, Accuracy TUG phases detection near 85%
Adame et al. [14]	2012	10 healthy (63.2 ± 10.1), 10 early stage PD (58.8 ± 9.5), 10 advanced stage PD (66.2 ± 4.8)	1 IMU, lower back	Estimation of TUG phases duration: small mean error, dynamic time warping
Milosevic et al. [15]	2013	3 PD, 4 healthy	Android Smartphone 3D-Acc, 3D-Gyro, 3D-Magn, chest	self-administered and automated TUG, rule-based
Reinfelder et al. [16]	2015	16 PD	2 IMU, SHIMMER 2R, 3D-Acc, 3D-Gyro, lateral side of both shoes	TUG phases recognition, Support Vector Machine, sensitivity: 81.80%
Nguyen et al. [17]	2017	4 females (67.8 ± 10.4) 8 males (66.6 ± 3.6) early stages PD	motion capture suit 17 IMU (3D-Acc/Gyro), 3D-Magn, each body segment	TUG activities recognition sensitivity: 97.6%, specificity: 92.7% modifications 100% accur. rule-based

**Table 2:** Characteristics of our included study population (*n* = 148) with minimum (min), maximum (max) and mean-value (mean), as well as the standard deviation (SD) of age in years, body weight in kg and body height in cm.

	Min	Max	Mean	SD
age (years)	70	87	75.22	3.83
weight (kg)	46.85	110.80	76.01	13.94
height (cm)	145.80	188.70	167.43	9.50

**Table 3:** Parameters for our classifiers: method, size and step-width of the sliding window, as well as the noise reduction filter and feature set. The abbreviations of the features are listed in the text. The used data for each feature are specified in brackets at the end of the line: Acceleration data (Acc), Gyroscope data (Gyro). The abbreviations HL and HN stand for hidden layer and hidden nodes. The cut-off frequency of the specific filters is $f_{c}$. AC, Auto Correlation; C, Correlation; SMA, Signal Magnitude Area.

	Classifier	Method	Window Size (s)	Step Width (s)	Filter	Feature-Set
(1)	State	Boosted Decision Trees	1.405	0.072	Low pass ($f_{c}$ = 6.1 Hz)	AC, C, Mean (Acc), RMS, SD, SE (Acc + Gyro)
(2)	Static	Multilayer Perceptrons (5 HL, 7 HN)	2.511	0.427	-	Mean, SMA (Acc), Pitch, AC, C (Acc + Gyro)
(3)	Dynamic	Multilayer Perceptrons (3 HL, 44 HN)	1.853	0.249	Gaussian σ=0.23	RMS (Acc), Pitch, AC, C, SMA, SD (Acc + Gyro)
(4)	Transition	Multilayer (4 HL, 40 HN) Perceptrons (4 HL, 40 HN)	1.135	0.073	Low pass ($f_{c}$ = 4.5 Hz)	RMS (Acc), Mean, SE (Gyro), AC, C, SMA, SD (Acc+Gyro), Pitch

**Table 4:** *F*1-scores of the classification methods for the different classifiers: Boosted Decision Trees (BDT), Multilayer Perceptrons (MLP).

Classifier	*F*1-Score (%)	Method (%)
State	96.6	BDT
Static	97.3	MLP
Dynamic	97.5	MLP
Transition	94.8	MLP

**Table 5:** Results of our classification model for static (sit, stand) and dynamic (walk, turn around) activities, as well as transitions (sit-to-stand, stand-to-sit).

Classifier	Activity	Recall	Precision	Accuracy	*F*1-Score
Static	Sit	0.93	0.96	0.96	0.95
	Stand	0.96	0.91	0.97	0.94
Dynamic	Turn around	0.78	0.83	0.99	0.81
	Walk	0.98	0.98	0.98	0.97
Transition	Sit-to-Stand	0.84	0.66	0.99	0.74
	Stand-to-Sit	0.94	0.56	0.99	0.70

**Table 6:** Included models and the resulting recognition accuracy, as well as the cumulative accuracy. The order of the activities for each model is listed in following terms: sit-to-stand (↑), Walk (W), Turn (T), stand-to-sit (↓).

#	Model	Accuracy $A_{j}$ (%)	Cum.Accuracy $A_{i}$ (%)
1	↑WTWT↓	15.37	15.37
2	↑↓WTWT↓	52.15	67.52
3	↑↓TWT↓	12.35	79.87
4	↑↓T↓	5.67	85.54
5	↑↓WT↓	4.34	89.88
6	↑TWT↓	2.34	92.22
7	↑↓WTWTW↓	1.67	93.89
8	↑WTWTW↓	1.33	95.22
9	↑WT↓	1.33	96.55

**Table 7:** Correlation coefficients of the Standard TUG duration (STUG) and Unsupervised TUG duration (UTUG) with other geriatric tests like for example the chair rising and gait speed of the Short Physical Performance Battery (SPPB). Significant results are marked with an asterisk (*).

	STUG		UTUG	
	r	*p*	r	*p*
Stair Climb Power Test	0.85	<0.01 *	0.52	<0.01 *
SPPB-Chair Rising Test	0.67	<0.01 *	0.28	<0.01 *
SPPB-Gait Speed	0.75	<0.01 *	0.36	<0.05 *
6-min Walk Test	−0.82	<0.01 **	−0.44	<0.01 *
